# Genomic epidemiology and antibiotic susceptibility profiling of uropathogenic *Escherichia coli* among children in the United States

**DOI:** 10.1128/msphere.00184-23

**Published:** 2023-08-15

**Authors:** Rauf Salamzade, Christi L. McElheny, Abigail L. Manson, Ashlee M. Earl, Nader Shaikh, Yohei Doi

**Affiliations:** 1 Infectious Disease & Microbiome Program, Broad Institute, Cambridge, Massachusetts, USA; 2 Division of Infectious Diseases, University of Pittsburgh School of Medicine, Pittsburgh, Pennsylvania, USA; 3 Children’s Hospital of Pittsburgh, University of Pittsburgh Medical Center, Pittsburgh, Pennsylvania, USA; 4 Fujita Health University School of Medicine, Aichi, Japan; Escola Paulista de Medicina/Universidade Federal de São Paulo, São Paulo, Brazil

**Keywords:** urinary tract infection, antibiotic resistance, pyelonephritis, *Escherichia coli*, genome analysis, P-fimbriae, virulence

## Abstract

*Escherichia coli* is the most common cause of urinary tract infections (UTIs) in children, and yet the underlying mechanisms of virulence and antibiotic resistance and the overall population structure of the species is poorly understood within this age group. To investigate whether uropathogenic *E. coli* (UPEC) from children who developed pyelonephritis carried specific genetic markers, we generated whole-genome sequence data for 96 isolates from children with UTIs. This included 57 isolates from children with either radiologically confirmed pyelonephritis or cystitis and 27 isolates belonging to the well-known multidrug-resistant sequence type ST131, selected to investigate their population structure and antibiotic resistance characteristics. We observed a UPEC population structure that is similar to those reported in adults. In comparison with prior investigations, we found that the full *pap* operon was more common among UPEC from pediatric cases of pyelonephritis. Further, in contrast with recent reports that the P-fimbriae adhesin-encoding *papGII* allele is substantially more prevalent in invasive UPEC from adults, we found *papGII* was common to both invasive and non-invasive UPEC from children. Among the set of ST131 isolates from children with UTIs, we found antibiotic resistance was correlated with known genetic markers of resistance, as in adults. Unexpectedly, we observed that *fimH*30, an allele of the fimbrial gene *fimH* often used as a proxy to type ST131 isolates into the most drug-resistant subclade C, was carried by some of the subclade A and subclade B isolates, suggesting that the *fimH*30 allele could confer a selective advantage for UPEC.

**IMPORTANCE:**

Urinary tract infections (UTIs), which are most often caused by *Escherichia coli*, are not well studied in children. Here, we examine genetic characteristics that differentiate UTI-causing bacteria in children that either remain localized to the bladder or are involved in more serious kidney infections. We also examine patterns of antibiotic resistance among strains from children that are part of *E. coli* sequence type 131, a group of bacteria that commonly cause UTIs and are known to have high levels of drug resistance. This work provides new insight into the virulence and antibiotic resistance characteristics of the bacteria that cause UTIs in children.

## INTRODUCTION

*Escherichia coli* is the most common cause of urinary tract infection (UTI), affecting all ages, including children. UTIs can be broadly classified into those involving the kidneys (pyelonephritis) and those that remain localized to the bladder (cystitis), which can only be differentiated based on radiological examination. Several virulence factors have been identified as contributing to the fitness of uropathogenic *E. coli* (UPEC), including toxins and proteases (1 – 6), iron acquisition systems (6 – 9), and adhesion fimbriae, which can be regulated by phase variation (6, 10 – 12). Early studies highlighted the carriage of a specific fimbria encoded by the pyelonephritis-associated pili (*pap*) operon in UPEC, as well as specific alleles of key genes within the operon, such as *papGII*, to be decisive for infections developing into pyelonephritis (11, 13, 14). In one of the few studies leveraging whole-genome sequencing to investigate the genetic traits that enhance *E. coli*’s ability to cause pyelonephritis or invasive infections, the *papGII* allele has been significantly associated with invasive UPEC in adults (9)*,* consistent with earlier findings that the *papGII* exhibits preferential binding to globosides, which are dominant in kidneys (15). This meta-study of 722 UPEC genomes from across multiple independent investigations, of which at least 80% were from adults, also showed that invasive UPEC are phylogenetically clustered within specific sublineages that tend to encode greater numbers of virulence genes, in particular those related to iron acquisition.

UPEC are becoming increasingly resistant to agents commonly used to treat UTI, such as fluoroquinolones, trimethoprim-sulfamethoxazole, and cephalosporins. While the use of fluoroquinolones is much less common among children, fluoroquinolone-resistant *E. coli* has been detected in this age group (16). UPEC are also exhibiting increased multidrug resistance (MDR), defined as exhibiting resistance to at least one antibiotic in three or more drug classes (17), in large part due to the emergence and dissemination of specific clonal lineages of MDR *E. coli*. Of these clonal lineages, sequence type (ST) 131 is considered the most successful worldwide and exhibits MDR (18). However, little is known about the population structure of ST131 *E. coli* causing UTI in children. Although previous reports suggested that ST131 tend to lack *papG* (19), one study identified specific sublineages of ST131 that encode *papGII* and associate with more severe UTIs (9). The objectives of this study were to identify alleles associated with pyelonephritis in children with UTI and to examine the population structure and antibiotic resistance of UTI-causing ST131 *E. coli* in children.

## RESULTS

### Isolates from children with pyelonephritis and cystitis are phylogenetically diverse

**Fig 1:**
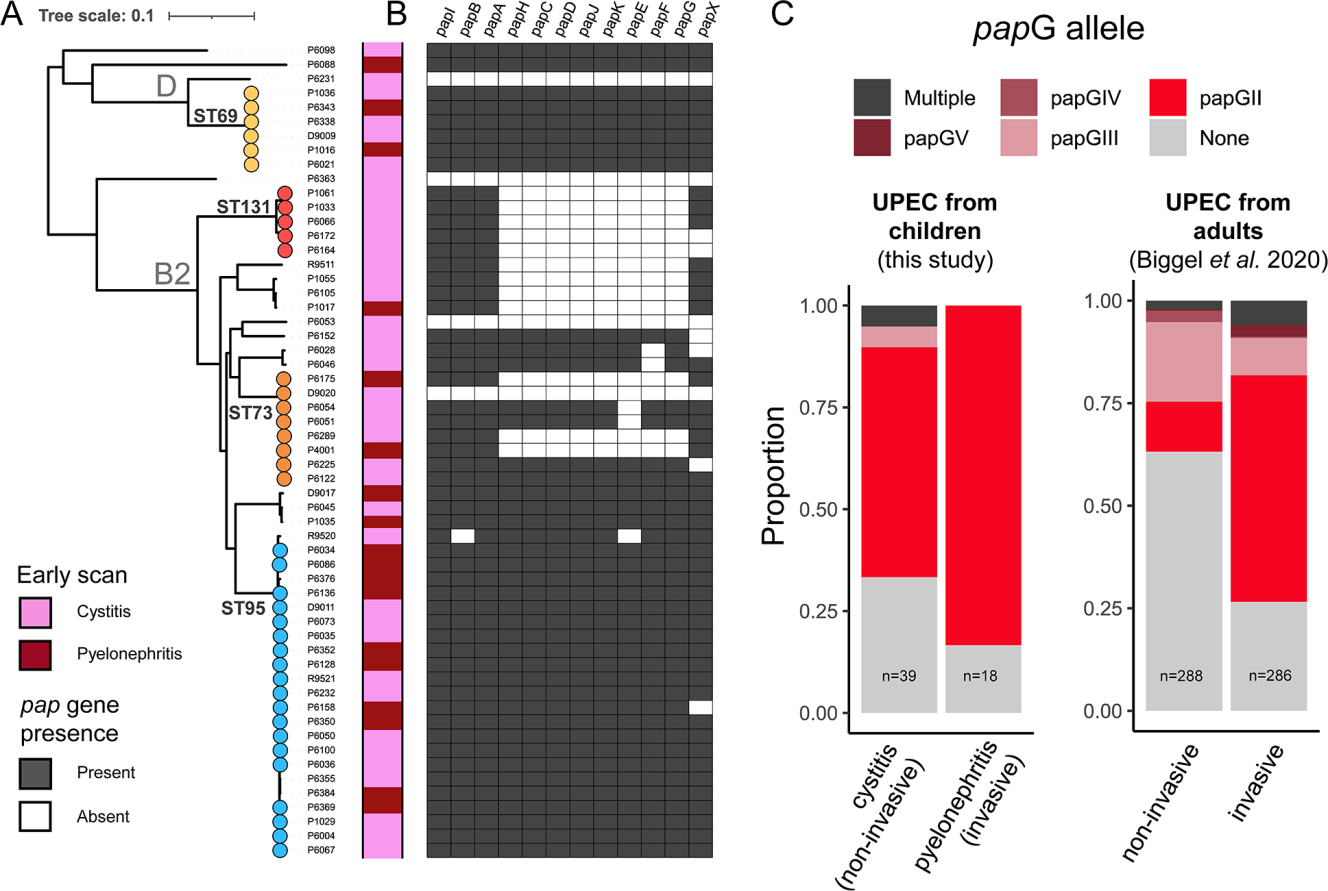
The presence of the full *pap* operon associates with pyelonephritis versus cystitis. (**A**) Maximum-likelihood phylogeny of the 57 isolates from patients with early scan diagnosis (Materials and Methods). Nodes in the phylogeny are colored by the four common sequence types we observed in the sample set. Phylogenetic clades corresponding to the common phylogroups B2 and D are marked. The colored track displays whether isolates were gathered from children diagnosed with cystitis (pink) or pyelonephritis (dark red). (**B**) Heatmap showing the presence (gray) or absence (white) of 12 *pap* operon genes in the sequencing data of each isolate. (**C**) *papG* carriage and alleles are illustrated for the 57 isolates from our study alongside UPEC from adults, extracted from supplementary files from the (9) study.

Of the 61 children with UTI who underwent a renal scan, 57 *E. coli* isolates yielded sufficient, high-quality sequencing data for inclusion in downstream genomic analyses (Table 1). To determine the evolutionary relationship among these isolates, we called single-nucleotide polymorphism (SNPs) against a common reference using Pilon (20), removed recombined regions predicted using ClonalFrameML (21), and constructed a maximum likelihood, SNP-based whole-genome phylogeny using RaxML (22). The majority of isolates (84%) belonged to phylogroup B2. To put the phylogenetic tree into context with known *E. coli* STs, we determined ST designations using ARIBA and the PubMLST database. Combined, the results of these analyses showed that isolates within this collection were phylogenetically diverse, representing at least 22 STs, a large fraction of which fell within ST95 (*n* = 19), ST73 (*n* = 8), ST69 (*n* = 6), and ST131 (*n* = 5) (Fig. 1; Fig. S1; Table S1), which are the same four STs noted previously to predominate among UPEC infections in adults (9). Although ST designations tended to track well with the placement of isolates within the phylogeny, we observed four isolates, labeled as ST2614 (*n* = 1), ST421 (*n* = 1), or “novel ST” (*n* = 2), closely clustered with other ST95 isolates within the SNP-based phylogeny. Due to their close phylogenetic relationship, we included these four isolates among ST95 members in downstream analyses (Fig. S1; Table S2).

**TABLE 1:** Demographic and clinical characteristics of included children^*a*^

	Children with *E. coli* UTI *N* = 57	Children with ST131 *E. coli* UTI *N* = 27
	No. (%)	No. (%)
Age in months at enrollment		
Median [IQR]	42.3 [9.7, 71.0]	10.8 [4.5, 50.6]
Gender—no. (%)		
Male	3 (5.3)	3 (11.1)
Female	54 (94.7)	24 (88.9)
Race—no. (%)		
White	29 (50.9)	12 (44.4)
Black	17 (29.8)	9 (33.3)
Other	11 (19.3)	6 (22.2)
Fever during the UTI event		
No	10 (17.5)	5 (18.5)
Yes	47 (82.5)	22 (81.5)
Method of urine collection—no. (%)		
Catheter	28 (49.1)	18 (66.7)
Clean catch	29 (50.9)	9 (33.3)
Leukocyte esterase—no. (%)		
Negative/trace	1 (1.8)	1 (3.7)
Small (+)	3 (5.3)	6 (22.2)
Moderate (++)	11 (19.3)	2 (7.4)
Large (+++)	42 (73.7)	18 (66.7)

^a^ Five children were included in both columns.

Layering on information about the disease status of the children from whom these *E. coli* isolates were obtained revealed no clear pattern between placement within the phylogeny and whether the isolate caused any specific disease. Isolates associated with a diagnosis of pyelonephritis (*n* = 18) or cystitis (*n* = 39) were distributed across the *E. coli* phylogeny (Fig. 1A). In addition, there was no significant overrepresentation of pyelonephritis or cystitis cases within any of the four highly represented clades, though none of the ST131 isolates were associated with pyelonephritis, and only slightly over half (55.6%; 10 of 18) of the pyelonephritis-causing isolates belonged to ST95.

### Association between carriage of the *pap* operon and pyelonephritis

Though the evolutionary relationship among isolates did not reveal any clear pattern to suggest an association with cystitis or pyelonephritis, the production of P-fimbriae encoded by the *pap* operon in *E. coli* has been associated with the development of pyelonephritis and renal scarring (11, 13, 14). To determine whether *pap* genes or any other virulence factors were significantly associated with pyelonephritis in children from this study, we first used ARIBA (23) together with the *Escherichia coli* virulence-associated gene database (EcVGDB) (9) to identify 639 distinct and functionally diverse virulence-associated genes in one or more of the 57 UPEC isolates (Table S3). We then applied a mixed effects model (FaST-LMM) implemented in pyseer (24, 25), which accounts for the population structure across our isolate set, to infer significant associations of virulence genes with UPEC from pyelonephritis cases (Table S4). Individually, no virulence genes were determined as significantly associated after accounting for multiple tests, including previously identified UPEC-associated virulence genes (6, 26) (Fig. S2). Because invasive UPEC were previously found to carry greater numbers of distinct virulence genes for certain functions, such as iron acquisition (9), we investigated whether virulence gene load was greater in pyelonephritis relative to cystitis-associated UPEC isolates across 14 classes of virulence factors (Fig. S3; Table S5). We found no significant differences in gene counts for any of the virulence classes between pyelonephritis and cystitis-related isolates.

While none of the *pap* genes were significantly associated with pyelonephritis individually, we found that the carriage of the full operon, consisting of 12 genes, was significantly associated with pyelonephritis (*P*-value = 0.0395). The full *pap* operon was found in 14 of 18 pyelonephritis-associated isolates (78%), while it was only found in 17 of 39 cystitis-associated isolates (44%) (Fig. 1A and B). It was carried by all ST69 isolates and nearly all isolates within the ST95 clade, in accordance with recent genomic analysis suggesting that the *pap* operon was obtained via a single pathogenicity island lateral transfer event in an ancestor of these clades (9). In contrast, most ST131 isolates lacked eight or nine of the twelve *pap* genes, including *papG*. The lack of *papG* was previously reported as a defining feature of ST131 (19); however, it was recently reported that *papGII* was associated with specific sublineages of invasive ST131 UPEC (9). Among the five ST131 UPEC in our study with diagnostic information for the UTI, all were related to incidents of cystitis.

All but three of the isolates from children with pyelonephritis (15/18; 83%) featured the *papGII* allele, which has previously been strongly associated with pyelonephritis development (9, 11) and shown to be important for binding to globoside glycosphingolipids, abundant in kidney cells (11, 15, 27) (Fig. 1C). However, *papGII* was also found in 62% of the isolates from children with cystitis, resulting in our inability to identify a significant association between *papGII* and pyelonephritis development within this data set. This is in contrast to recent findings from a genome-wide association study (GWAS), which found *papGII* as the most significant virulence factor associated with invasive UPEC (9). To further investigate, we extracted information for *papG* allele carriage among 574 UPEC from cases of invasive or non-invasive UPEC in adults from the study by Biggel et al. Only six of the remaining 148 UPEC in their study were clearly designated as being isolated from children. Our investigation confirmed that *papGII* carriage is larger in non-invasive cystitis cases in children compared to non-invasive cases in adults (*P*-value = 2.599e-9; one-sided Fisher’s exact test) (Fig. 1C). Of note, *papGII* presence was not always concordant with carriage of the full *pap* operon.

### Extensive drug resistance within pediatric *E. coli* ST131

ST131 *E. coli* are notorious for their ability to resist multiple antimicrobials as well as for their rapid global spread (28). ST131 has further been subdivided into the major subclades A, B, and C (not to be confused with species-wide phylogroup designations of *E. coli*) (29). Subclade C in particular has been shown to exhibit intrinsic resistance to several clinically relevant drugs, including β-lactams and fluoroquinolones (18, 28).

**Fig 2:**
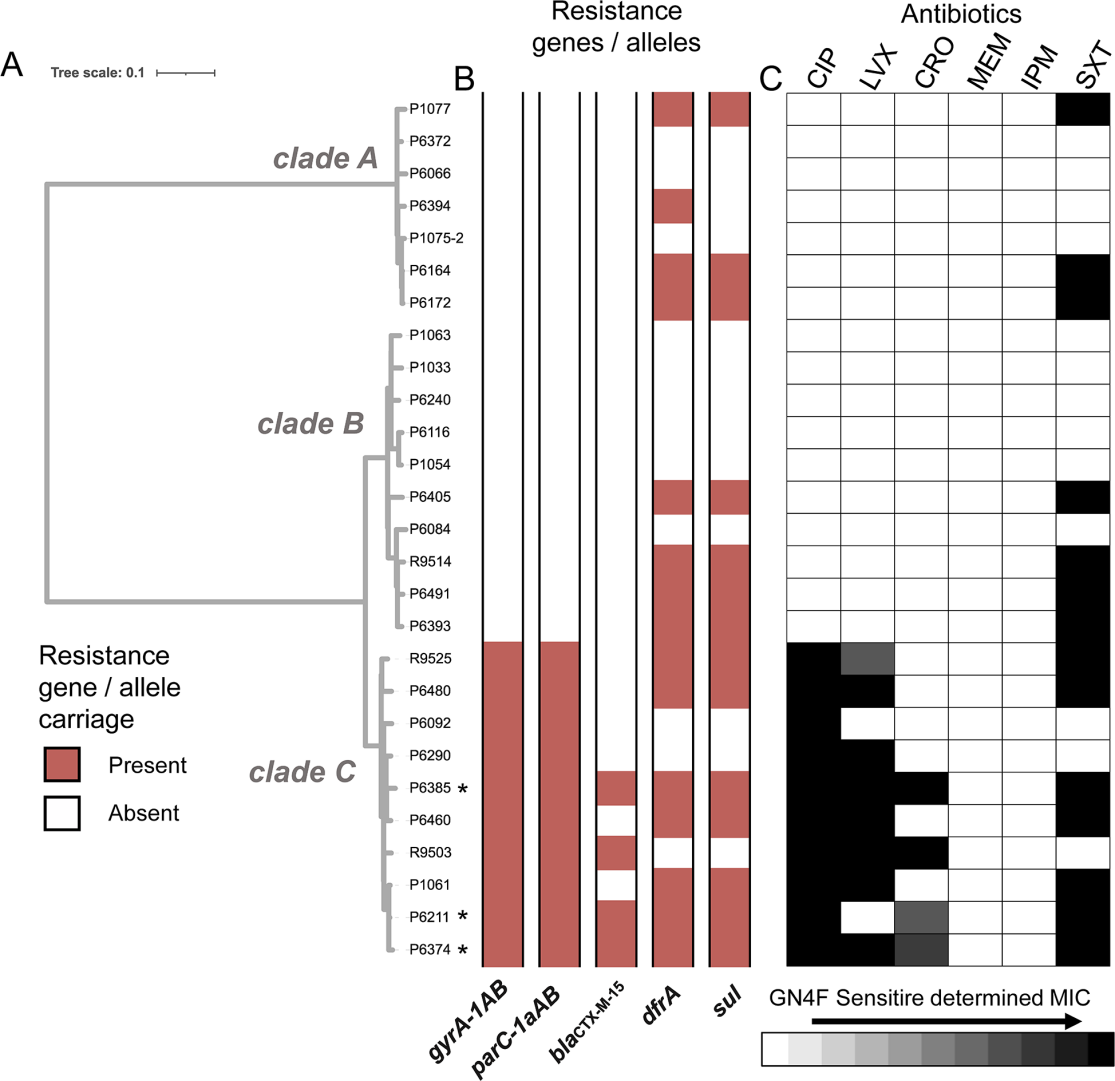
Antibiotic resistance among ST131 isolates. (**A**) Maximum-likelihood phylogeny of 27 ST131 isolates. Asterisks (*) denote MDR isolates. (**B**) Colored tracks depict the presence of fluoroquinolone resistance-associated alleles for the core genes *gyrA* and *parC*, as well as the carriage of *bla*_CTX-M-15_, *sul*, and *dfrA*. (**C**) Heatmap depicting the degree of resistance exhibited by each isolate to six clinically relevant antibiotics using the Sensititre Gram Negative GN4F assay. Lighter colored cells indicate that the isolate is more susceptible to a particular antibiotic at the lowest dosage, whereas a black cell indicates that the isolate exhibits high levels of resistance to even the largest dosage tested. Antibiotic abbreviations: CIP, ciprofloxacin; LVX, levofloxacin; CRO, ceftriaxone; MEM, meropenem; IPM, imipenem; SXT, trimethoprim-sulfamethoxazole.

Of the total set of 48 *E. coli* isolates suspected of belonging to the ST131 lineage, 27 were confirmed by genomic typing (Table 1). To construct a more highly resolved phylogeny to examine the population structure of ST131 in children and its relationship to antibiotic resistance, we constructed a maximum likelihood, SNP-based whole genome phylogeny of only ST131 isolates (Fig. 2). To define subclade membership, we also searched sequencing reads from each isolate for subclade-specific primer sequences (30). As expected, clade membership corresponded to deep divisions within the ST131-specific phylogeny (Fig. 2); 7 isolates (26%) were part of ST131 subclade A, 10 (37%) belonged to subclade B, and 10 (37%) belonged to subclade C. Despite some relatively close relationship, there was little evidence for clonal spread among these ST131 isolates; on average, isolates were separated from one another by 112 SNPs with nearest neighbors separated by an average of 51 SNPs. Unexpectedly, we observed *fimH*30, an allele of the fimbrial gene *fimH* often used as a proxy for ST131 isolates into subclade C (31, 32), carried by three members of subclade A and four members of subclade B isolates (Fig. S4), suggesting that this typing allele lacks specificity and may be subject to horizontal exchange across subclade boundaries. We confirmed this unusual subclade distribution of *fimH* by further checking that ST131 subclade alleles for *parC* and *gyrA* (28) aligned with our phylogenomic model as well as confirming the absence of ambiguity in base calls within *fimH*30 (Fig. S4).

Understanding the extent of antibiotic resistance among ST131 UPEC in children could better inform which treatments are necessary and appropriate, in particular because early antibiotic exposure has been associated with potential long-term effects (33, 34). To examine the relationship between phenotypic drug resistance among ST131 isolates and the underlying genetic features determining resistance, we first examined the susceptibility results for 23 drugs or combinations of agents (Fig. 2; Table S6). We focused on resistance to six antibiotics, which are commonly prescribed for UTIs at clinics, including two last-resort drugs, representative of four antibiotic classes—fluoroquinolones, cephalosporins, carbapenems, and antifolates. Among the set of 27 ST131 UPEC, three isolates were identified as MDR, exhibiting resistance to at least one antibiotic from three of the four antibiotic classes. As expected, all three multidrug-resistant isolates were within subclade C, showing resistance to fluoroquinolones, cephalosporins, and antifolates. Further, all subclade C isolates exhibited resistance to ciprofloxacin (*n* = 10), 80% of subclade C isolates demonstrated resistance to levofloxacin (*n* = 8), and 40% of subclade C isolates had resistance to ceftriaxone (*n* = 4). Resistance to trimethoprim-sulfamethoxazole was distributed across all three subclades, but also had the highest incidence within subclade C (*n* = 3 [43%] of subclade A; *n* = 4 [40%] of subclade B; *n* = 7 [70%] of subclade C). No ST131 isolates were resistant to carbapenems (meropenem and imipenem), which are last-resort antibiotics for the treatment of drug-resistant infection.

For six of the most clinically relevant antibiotics or combinations, we compared the phenotypic results to the carriage of antimicrobial resistance (AMR) genes identified in our genomic sequencing of isolates in order to understand whether known genetic determinants explain the observed phenotypes (Fig. 2; Tables S7 and S8). For each of these drugs, we observed full concordance between genotype and phenotype. All 10 strains exhibiting resistance to a fluoroquinolone (levofloxacin or ciprofloxacin) carried the *parC_1aAB* and *gyrA_1AB* mutations in their quinolone resistance-determining regions (QRDRs), which have been shown previously to lead to fluoroquinolone resistance and have been reported as a core feature in the emergence of ST131 subclade C (28). Of the four strains exhibiting resistance to ceftriaxone, an expanded-spectrum cephalosporin, all four carried the extended-spectrum β-lactamase gene *bla*_CTX-M-15_. Of the 14 isolates that exhibited resistance to trimethoprim-sulfamethoxazole, all carried the *sul1* gene encoding a dihydropteroate synthase, which confers sulfamethoxazole resistance, together with either *dfrA*12 or *dfrA*17, which encode dihydrofolate reductases previously shown to confer trimethoprim resistance (Table S8).

## DISCUSSION

We found that UPEC from children with pyelonephritis were more likely to have the full suite of coding genes in the *pap* operon responsible for the production of P-fimbriae, which has long been associated with pyelonephritis in UPEC (9, 11, 14). Many studies have found that P-fimbriae contribute to UPEC virulence and the development of pyelonephritis and related diseases (14, 35 – 37). Here, we provide additional support for these viewpoints by using genomic analysis to show that the carriage of the full *pap* machinery is more prevalent among UPEC isolates collected from children who were diagnosed with pyelonephritis as compared to UPEC collected from cases of more acute cystitis. Additionally, while the rates of UPEC from pyelonephritis cases with the full suite of *pap* machinery (78%) were roughly equivalent to the rate of UPEC with P-fimbriae observed in a study by Kallenius et al. (91%) (14), we observed a substantially higher rate of UPEC from cystitis cases carrying the *pap* machinery (44%), when compared to the percentage of UPEC from such cases reported to feature P-fimbriae in the aforementioned study (19%). This discrepancy could potentially be explained by host factors influencing immunity to infection (38), differences in sampling and diagnosis between the two studies, or epigenetic phase variation in the regulation of the *pap* operon among the UPEC isolates (39 – 41), for which Kallenius et al. used agglutination assays that would only detect active P-fimbriae to assess their presence oin UPEC from children. Specifically, pyelonephritis and cystitis cannot be accurately differentiated based on the child’s signs or symptoms or any laboratory tests; many children with cystitis have fever, abdominal/back pain, and/or an elevated inflammatory marker. As such, previous studies may have incorrectly classified children being studied. A strength of our study is that we used renal scans, the current gold standard for the diagnosis of pyelonephritis, to categorize the children in the study.

Investigations around P-fimbriae have also identified several classes of the gene coding for their tip adhesin, *papG*, which have been shown to differentially bind to specific glycolipids found on host cells (11, 15). In particular, the *papGII* allele has been closely associated with pyelonephritis development and shown to bind to globoside molecules, which are abundantly found on mammalian kidney cells (11, 15, 27, 42). *papGII* has also been shown to induce changes in host gene expression (43). In a recent GWAS, Biggel et al. associated genomic markers with invasive UPEC, mostly from adults, and once again highlighted *papGII* as a key factor associated with the diagnosis of more severe UTIs (9). However, we did not observe this same association in our data set; we found that a majority of the isolates in our study that carried the *papG* gene featuring the *papGII* allele were associated with cystitis. While these results suggest potential differences in *papGII* prevalence in UPEC from children compared to UPEC from adults, they could also be confounded by differences in sampling location and differences in how strains were associated with UTI severity between the studies. Our results may have also differed because we strictly compared UPEC associated with pyelonephritis with those associated with cystitis based on radiological diagnosis, unlike Biggel et al., who investigated differences between the broader categorizations of invasive and non-invasive UPEC.

Within the data set of *E. coli* gathered from the 57 children who were diagnosed with either pyelonephritis or cystitis as well as the set of 27 ST131 isolates, we also examined the population structure of UTI-causing *E. coli*. Although specific STs were highly represented, we found that the *E. coli* strains were diverse in children. The high count of SNPs separating isolates suggested that most UPEC isolates did not share recent ancestry and were thus not related to clonal outbreaks in hospitals but rather originated from diverse *E. coli* reservoirs outside the hospital, such as in food or the host’s own gastrointestinal tract. This suggests ongoing selection for P-fimbriae in UPEC involved in pyelonephritis, as others have speculated (9, 44, 45).

In one of the only studies of pediatric ST131 UPEC isolates to date, we found similar patterns of resistance as observed in other studies and high concordance between microbiological resistance and known resistance determinants. We found that 56% of isolates were resistant to trimethoprim-sulfamethoxazole and 37% were resistant to ciprofloxacin, in accordance with previous reports of 30–60% of ST131 strains resistant to fluoroquinolones (46). As expected, all 10 ST131 subclade C isolates were resistant to ciprofloxacin, a hallmark of this epidemic subclade due to its QRDR mutations within *gyrA* and *parC*. Sequence-based profiling confirmed the presence of these resistance-contributing mutations in subclade C isolates, and we were similarly able to relate known resistance genotypes of ST131 isolates with susceptibility phenotyping for other antibiotics such as ceftriaxone and trimethoprim-sulfamethoxazole. Given that fluoroquinolones are rarely used in children, this suggests that ST131 subclade C isolates may be spreading in them through either household transmission or yet unidentified collateral selective pressure.

We found that *fimH*30, the *fimH* allele that is tightly associated with ST131 subclade C (31), was carried by three subclade A and four subclade B isolates, suggesting the effects of recombination, and possibly a biological advantage for the carriage of this allele outside of clade C. Recombination has previously been reported to affect *fimH,* which encodes a fimbrial adhesin (47), but is likely underappreciated due to the common usage of *fimH* for typing ST131 strains into clades (32) (Fig. S2). Importantly, we did not observe the clade A-associated *fimH*41 and the clade B-associated *fimH*22 alleles to occur outside of their associated clades. In alignment with recent experimental findings that *fimH*30 can enhance biofilm formation and host cell adhesion relative to other variants of the *fimH* gene (48), our observations that the allele has likely been laterally acquired in strains belonging to ST131 subclades A and B further build support that it offers a selective advantage for UPEC.

Limitations of our study included convenience sampling and a relatively small sample size. Strengths include the prospective design, detailed clinical and laboratory characterization of each patient, use of a dimercaptosuccinic acid (DMSA) renal scan to differentiate pyelonephritis versus cystitis, and a small timescale (2012–2016) in which samples were collected, limiting confounding effects from evolutionary trends.

In conclusion, using whole-genome sequencing, we provide further support for the association between the carriage of the full, 12-gene *pap* operon and pyelonephritis. In agreement with previous work, we observed that the *papGII* allele was found in most isolates resulting in pyelonephritis; however, in our study, *papGII* was also found in a majority of cystitis-causing isolates. *papGII* was also observed within multiple incomplete *pap* operons, highlighting the importance of considering context and full operon presence. Additionally, we observed a diverse representation of ST131 UPEC isolates associated with infections in children, for which antibiotic susceptibility phenotyping largely corresponded to expectations based on literature and genotypic markers. Interestingly, we observed evidence of inter-clade recombination of *fimH*30, thought to be a key marker of subclades within ST131, suggesting that care must be taken when using *fimH*30 to infer clade membership. We also found that UPEC in childhood UTIs exhibit a similar population structure as UPEC from adult infections (9) and that 37% of ST131 isolates from UPEC in children belonged to the highly drug-resistant subclade C.

## MATERIALS AND METHODS

### Isolate collection and drug susceptibility testing

#### Selection of samples for comparisons between strains isolated from pyelonephritis versus cystitis cases

Urine cultures were obtained from children presenting with UTI, who were enrolled in two previously described studies (49, 50) conducted in the emergency room or outpatient clinical offices at five centers in the United States. The study was approved by the Institutional Review Boards at the participating centers. *E. coli* was identified from urine cultures at the clinical microbiology laboratories, and a single colony was stored in glycerol at −80°C and subsequently used for this study. All children participating in these studies presented with signs and symptoms of a UTI, had pyuria on urinalysis, and were eventually diagnosed with a UTI based on their urine culture results. Occurrence of fever during the UTI episode was recorded. In one of the studies (49), we offered a renal scan within 2 wk of the diagnosis of UTI to all children whose parents consented to this procedure. Of the 111 children with UTI in that study, 82 parents agreed to an early renal scan. Of these, 61 had isolates of *E. coli* that were stored. DNA from these 61 isolates, which represent a convenience sample from children with UTI, was sent to the Broad Institute for whole-genome sequencing (Table S1).

#### Selection of samples for analysis of population structure of ST131

We screened all 359 *E. coli* isolates in the aforementioned two studies for ST131 using a published multiplex PCR method, which collectively captured subclades A, B, and C within ST131 (51). Of the 359 *E. coli* isolates in both studies, 47 were suspected of belonging to ST131 as they screened positive for O-antigen types 16 or 25 by PCR, serotypes often associated with ST131 *E. coli* (19). One additional isolate that was not predicted to belong to ST131 based on serotype but exhibited resistance to multiple antimicrobials (gentamycin, ciprofloxacin, and trimethoprim-sulfamethoxazole), common for ST131, was also selected. DNA from these 48 isolates was sent to the Broad Institute for whole-genome sequencing (WGS; Table S1). Of note, six isolates were selected for both aims (i.e., they were suspected to belong to ST131 and also had an early renal scan to localize the site of UTI). Thus, a total of 103 isolates were sequenced between the two aims. For the 48 suspected ST131 isolates, we centrally determined the minimum inhibitory concentrations (MICs) of 23 agents by the broth microdilution method using commercially available dry plates (Sensititre GN4F, Thermo) (Table S6). Susceptibility was interpreted using the CLSI guidelines (M100-S28).

### Genome sequencing, assembly, and annotation

#### Extraction of genomic DNA and WGS

Genomic DNA was purified using DNeasy Blood and Tissue kits (Qiagen) and sent to the Broad Institute. Sequencing libraries were generated from 2 ng of input DNA using the Nextera XT DNA Library Preparation kit (Illumina) according to the manufacturer’s recommended protocol. Libraries were sequenced on an Illumina HiSeq 2500 with 151 bp paired-end reads.

#### Quality control and processing of sequencing data

Sequencing data were processed using Picard tools (https://broadinstitute.github.io/picard/). All but one of the samples had sufficient reads; the remaining sample (P6076) was removed from downstream analysis. After demultiplexing, adapters were removed using TrimGalore with default “--nextera” settings (52), and quality filtering was performed using Trimmomatic with lenient settings (leading:3, trailing:3, sliding-window:4:15) (53). After adapter removal and quality filtering, the average chromosome-wide sequencing coverage ranged from 56X to 514X, with a median of 174X, based on alignment to the reference *E. coli* Eco889 genome (GCF_001663475.1).

To assess the presence of sequence contamination from other genera, sequencing data were then aligned to a Centrifuge (54) index of archaeal, bacterial, and viral sequences from RefSeq (built in April 2018). Two isolates that had >10% of their reads aligned to a genus other than *Escherichia* or *Shigella* were excluded from analysis. To assess the presence of contamination due to multiple strains from within the same genus, we constructed draft assemblies using SPAdes (v 3.12.0) (55) and examined the length of the total genome assembled. Four samples had genome lengths exceeding the size of a typical *E. coli*’s genome (>6 Mbp), thus likely indicating additional contamination.

All seven isolates excluded from analysis are noted in Table S1, along with their reasons for exclusion. Four of these excluded isolates were among those selected for analysis of pyelonephritis versus cystitis, and four were among those selected for analysis of ST131, including one excluded isolate that was shared between the two studies. This left 57 isolates remaining for the cystitis versus pyelonephritis analysis and 44 for the ST131 analysis.

#### Sequence typing

ARIBA (23) was used with default settings to determine STs for isolates using the pubMLST/EnteroBase MLST database (56). Isolates were further identified as being members of ST clades by examination of the phylogeny (Fig. S1). Phylogroups were assigned based on available mappings of STs to phylogroups (9) together with phylogenomic inference. Computational predictions of serotype were made using the EcOH database (57) with SRST2 (58).

ST131 isolates were further categorized into their respective subclades by searching for previously published, subclade-specific primer sets (30, 59, 60) for subclades in sample sequencing read sets. Of the 44 isolates with high-quality sequencing data originally predicted to represent ST131 based on O antigen or an MDR profile, 19 were not predicted to be ST131 from MLST analysis and excluded, leaving 25 isolates for downstream analyses of ST131. We additionally included two isolates originally selected for comparisons of cystitis versus pyelonephritis, which were identified by MLST analysis as ST131, for a total of 27 isolates for analysis of the ST131 clade.

#### Allelic typing of fimH, gyrA, and parC in ST131 isolates

Genetic sequences distinguishing specific *fimH*, *gyrA*, *parC,* and CTX-M alleles were gathered (29) (https://github.com/BeatsonLab-MicrobialGenomics/VFDB/tree/master/fimH_parC_gyrA; https://github.com/BeatsonLab-MicrobialGenomics/VFDB/tree/6332a31d6ceeaafe5c617965ad9c0a2e56d077f9/additional_databases) and used to construct individual databases for sequence typing using ARIBA, with default settings. Following ARIBA analysis, we manually inspected allelic calls for validity.

Because *fimH*30, an allelic variant used as a marker of ST131 subclade C, was also observed outside of subclade C, we performed additional analysis to confirm this unexpected distribution, including (i) verifying that alleles of alternative marker genes used for subclade typing of ST131, such as *gyrA* and *parC*, were in agreement with subtype designations based on whole-genome phylogeny (gyrA-1A for subclade A, gyrA-1a for subclade B, gyrA-1AB for subclade C, parC-1b for subclade A, parC-1 for subclade B, and parC-1aAB for subclade C) and (ii) confirming that any base call ambiguity did not affect *fimH*30 by performing alignment of sequencing reads to a *fimH* reference and examining variants using Pilon (20).

#### Detection of AMR and virulence genes

ARIBA (23) was used with default settings to identify known AMR genes cataloged in the CARD database (downloaded August 2018) (61) and virulence factors from EcVGDB (9). Because EcVGDB does not exist as an integrated database for ARIBA, we manually created an ARIBA database, which resulted in 70 of 1,368 entries being removed due to either length or because the sequences did not resemble a coding gene as expected. ARIBA was run directly on processed, unassembled sequencing data. AMR and virulence genes were considered present in a sample if the local, targeted assembly of reads homologous to the gene produced by ARIBA had at least 90% identity and 80% coverage of the reference gene.

#### Allelic typing of papG

The EcVGDB database (9), which featured five distinct alleles of *papG*, was used with ARIBA to directly type *papG* alleles in our isolates as described earlier. Supplementary tables from Biggel et al., 2020 (9), which used and introduced the EcVGDB, were queried for *papG* carriage and alleles in UPEC from adults.

### Comparative analysis

#### Phylogenetic analysis

Reads were mapped to the ST131 reference strain *E. coli* Eco889 (GCF_001663475.1) using bwa version 0.7.17 (62). This reference was selected because its genome is complete, it belongs to ST131, and it was isolated from a urine sample (63). Samtools and Picard were used to sort, index, and mark duplicates in the resulting alignment files (64). Pilon version 1.22 (20) was used to identify variants using default settings. Variant loci with a mean mapping quality <10 were excluded. ClonalFrameML was used to predict and remove recombined regions (20), resulting in a core alignment with 12,004 SNP sites. RAxML was used with the GTRCAT model to construct a maximum-likelihood phylogeny with 1000 bootstrap replicates (22). From this larger phylogeny, a subset was extracted using PareTree (65), corresponding to the 57 isolates from patients who underwent an early scan.

Applying this same protocol, we generated a separate maximum-likelihood phylogeny for the ST131 clade. Based on our large tree, we selected all strains that were part of the clade containing ST131, which included the 27 expected ST131 isolates plus an additional ST2279 isolate, which was nested among the ST131 isolates. The core alignment used for this phylogeny construction contained 2,790 SNP sites. The ST2279 strain was removed from downstream visualization.

#### Testing for genotypic association with pyelonephritis development

The carriage of specific virulence genes was tested for enrichment or depletion within the isolates collected from patients diagnosed with pyelonephritis versus cystitis using the pyseer package (24). The presence or absence of virulence genes was encoded as binary genotypic traits, and the diagnosis of pyelonephritis was treated as a binary phenotype. Association analysis was carried out using a mixed effects model (FaST-LMM) (25) with a kinship matrix constructed from the whole-genome phylogeny after recombination removal to account for the population structure of our sample set and to overcome the influence by lineage effects. Multiple hypothesis testing correction was performed using the count_patterns.py script provided within pyseer across all individual virulence genes. A separate, targeted test was performed for the association of the full *pap* operon with pyelonephritis development.

To assess differences in virulence gene load for different virulence classes between UPEC from pyelonephritis versus cystitis, we used a permutation-based test. Briefly, for each virulence class (Table S4), we performed 10,000 simulations, where designations for UPEC as cystitis or pyelonephritis associated were shuffled. The resulting median gene counts for pyelonephritis and cystitis isolates in simulations were compared to the actual observed values. If the difference for a simulation was greater than or equal to the actual observed difference, a counter was incremented. An empirical *P*-value was then calculated for the virulence class based on dividing the counter by the number of simulations, with a pseudocount of one added to the numerator and denominator to avoid assigning a *P*-value of zero. The false discovery rate across virulence classes was controlled using the Benjamini–Hochberg procedure.

## Data Availability

Sequencing read data was submitted to NCBI’s SRA database under BioProject ID PRJNA448593.

## SUPPLEMENTAL MATERIAL

Supplemental Figures and Table msphere.00184-23-s0001.pdf Figures S1 to S7 and Table S8.

Supplemental Tables msphere.00184-23-s0002.xlsx Tables S1-S7.

ASM does not own the copyrights to Supplemental Material that may be linked to, or accessed through, an article. The authors have granted ASM a non-exclusive, world-wide license to publish the Supplemental Material files. Please contact the corresponding author directly for reuse.

## References

[1] Dhakal BK , Mulvey MA . 2012. The UPEC pore-forming toxin α-hemolysin triggers proteolysis of host proteins to disrupt cell adhesion, inflammatory, and survival pathways. Cell Host Microbe 11:58–69. doi:10.1016/j.chom.2011.12.003 22264513 PMC3266558

[2] Nagy G , Altenhoefer A , Knapp O , Maier E , Dobrindt U , Blum-Oehler G , Benz R , Emody L , Hacker J . 2006. Both alpha-haemolysin determinants contribute to full virulence of uropathogenic Escherichia coli strain 536. Microbes Infect 8:2006–2012. doi:10.1016/j.micinf.2006.02.029 16787757

[3] Guyer DM , Radulovic S , Jones F-E , Mobley HLT . 2002. Sat, the secreted autotransporter toxin of uropathogenic Escherichia coli, is a vacuolating cytotoxin for bladder and kidney epithelial cells. Infect Immun 70:4539–4546. doi:10.1128/IAI.70.8.4539-4546.2002 12117966 PMC128167

[4] Maroncle NM , Sivick KE , Brady R , Stokes F-E , Mobley HLT . 2006. Protease activity, secretion, cell entry, cytotoxicity, and cellular targets of secreted autotransporter toxin of uropathogenic Escherichia coli. Infect Immun 74:6124–6134. doi:10.1128/IAI.01086-06 16954394 PMC1695523

[5] Heimer SR , Rasko DA , Lockatell CV , Johnson DE , Mobley HLT . 2004. Autotransporter genes pic and tsh are associated with Escherichia coli strains that cause acute pyelonephritis and are expressed during urinary tract infection. Infect Immun 72:593–597. doi:10.1128/IAI.72.1.593-597.2004 14688142 PMC343984

[6] Subashchandrabose S , Mobley HLT. 2016. Virulence and fitness determinants of uropathogenic *Escherichia coli* , p. 235–261. In Urinary Tract Infections. ASM Press, Washington, DC, USA. 10.1128/microbiolspec.UTI-0015-2012 PMC4566162 26350328

[7] Henderson JP , Crowley JR , Pinkner JS , Walker JN , Tsukayama P , Stamm WE , Hooton TM , Hultgren SJ , Van Nhieu GT . 2009. Quantitative metabolomics reveals an epigenetic blueprint for iron acquisition in uropathogenic Escherichia coli. PLoS Pathog 5:e1000305. doi:10.1371/journal.ppat.1000305 19229321 PMC2637984

[8] Hagan EC , Mobley HLT . 2009. Haem acquisition is facilitated by a novel receptor Hma and required by uropathogenic Escherichia coli for kidney infection. Mol Microbiol 71:79–91. doi:10.1111/j.1365-2958.2008.06509.x 19019144 PMC2736550

[9] Biggel M , Xavier BB , Johnson JR , Nielsen KL , Frimodt-Møller N , Matheeussen V , Goossens H , Moons P , Van Puyvelde S . 2020. Horizontally acquired papGII-containing pathogenicity islands underlie the emergence of invasive uropathogenic Escherichia coli lineages. Nat Commun 11:5968. doi:10.1038/s41467-020-19714-9 33235212 PMC7686366

[10] Connell I , Agace W , Klemm P , Schembri M , Mărild S , Svanborg C . 1996. Type 1 fimbrial expression enhances Escherichia coli virulence for the urinary tract. Proc Natl Acad Sci U S A 93:9827–9832. doi:10.1073/pnas.93.18.9827 8790416 PMC38514

[11] Lane MC , Mobley HLT . 2007. Role of P-fimbrial-mediated adherence in pyelonephritis and persistence of uropathogenic Escherichia coli (UPEC) in the mammalian kidney. Kidney Int 72:19–25. doi:10.1038/sj.ki.5002230 17396114

[12] Stærk K , Khandige S , Kolmos HJ , Møller-Jensen J , Andersen TE . 2016. Uropathogenic Escherichia coli express type 1 fimbriae only in surface adherent populations under physiological growth conditions. J Infect Dis 213:386–394. doi:10.1093/infdis/jiv422 26290608

[13] Edén CS , Hansson HA . 1978. Escherichia coli Pili as possible mediators of attachment to human urinary tract epithelial cells. Infect Immun 21:229–237. doi:10.1128/iai.21.1.229-237.1978 361565 PMC421981

[14] Källenius G , Möllby R , Svenson SB , Helin I , Hultberg H , Cedergren B , Winberg J . 1981. Occurrence of P-fimbriated Escherichia coli in urinary tract infections. Lancet 2:1369–1372. doi:10.1016/s0140-6736(81)92797-5 6171697

[15] Strömberg N , Nyholm PG , Pascher I , Normark S . 1991. Saccharide orientation at the cell surface affects glycolipid receptor function. Proc Natl Acad Sci U S A 88:9340–9344. doi:10.1073/pnas.88.20.9340 1681550 PMC52710

[16] Logan LK , Medernach RL , Rispens JR , Marshall SH , Hujer AM , Domitrovic TN , Rudin SD , Zheng X , Qureshi NK , Konda S , Hayden MK , Weinstein RA , Bonomo RA . 2019. Community origins and regional differences highlight risk of Plasmid-mediated fluoroquinolone resistant Enterobacteriaceae infections in children. Pediatr Infect Dis J 38:595–599. doi:10.1097/INF.0000000000002205 30281548 PMC6440871

[17] Magiorakos A-P , Srinivasan A , Carey RB , Carmeli Y , Falagas ME , Giske CG , Harbarth S , Hindler JF , Kahlmeter G , Olsson-Liljequist B , Paterson DL , Rice LB , Stelling J , Struelens MJ , Vatopoulos A , Weber JT , Monnet DL . 2012. Multidrug-resistant, extensively drug-resistant and pandrug-resistant bacteria: an international expert proposal for interim standard definitions for acquired resistance. Clin Microbiol Infect 18:268–281. doi:10.1111/j.1469-0691.2011.03570.x 21793988

[18] Mathers AJ , Peirano G , Pitout JDD . 2015. Escherichia coli ST131: the quintessential example of an international multiresistant high-risk clone. Adv Appl Microbiol 90:109–154. doi:10.1016/bs.aambs.2014.09.002 25596031

[19] Nicolas-Chanoine M-H , Bertrand X , Madec J-Y . 2014. Escherichia coli ST131, an intriguing clonal group. Clin Microbiol Rev 27:543–574. doi:10.1128/CMR.00125-13 24982321 PMC4135899

[20] Walker BJ , Abeel T , Shea T , Priest M , Abouelliel A , Sakthikumar S , Cuomo CA , Zeng Q , Wortman J , Young SK , Earl AM . 2014. Pilon: an integrated tool for comprehensive microbial variant detection and genome assembly improvement. PLoS One 9:e112963. doi:10.1371/journal.pone.0112963 25409509 PMC4237348

[21] Didelot X , Wilson DJ . 2015. Clonalframeml: efficient inference of recombination in whole bacterial genomes. PLoS Comput Biol 11:e1004041. doi:10.1371/journal.pcbi.1004041 25675341 PMC4326465

[22] Stamatakis A . 2006. RAxML-VI-HPC: maximum likelihood-based phylogenetic analyses with thousands of taxa and mixed models. Bioinformatics 22:2688–2690. doi:10.1093/bioinformatics/btl446 16928733

[23] Hunt M , Mather AE , Sánchez-Busó L , Page AJ , Parkhill J , Keane JA , Harris SR . 2017. ARIBA: rapid antimicrobial resistance genotyping directly from sequencing reads. Microb Genom 3:e000131. doi:10.1099/mgen.0.000131 29177089 PMC5695208

[24] Lees JA , Galardini M , Bentley SD , Weiser JN , Corander J . 2018. Pyseer: a comprehensive tool for microbial pangenome-wide association studies. Bioinformatics 34:4310–4312. doi:10.1093/bioinformatics/bty539 30535304 PMC6289128

[25] Lippert C , Listgarten J , Liu Y , Kadie CM , Davidson RI , Heckerman D . 2011. FaST linear mixed models for genome-wide association studies. Nat Methods 8:833–835. doi:10.1038/nmeth.1681 21892150

[26] Liu B , Zheng D , Jin Q , Chen L , Yang J . 2019. VFDB 2019: a comparative pathogenomic platform with an interactive web interface. Nucleic Acids Res 47:D687–D692. doi:10.1093/nar/gky1080 30395255 PMC6324032

[27] Dodson KW , Pinkner JS , Rose T , Magnusson G , Hultgren SJ , Waksman G . 2001. Structural basis of the interaction of the pyelonephritic E. coli adhesin to its human kidney receptor. Cell 105:733–743. doi:10.1016/s0092-8674(01)00388-9 11440716

[28] Pitout JDD , DeVinney R . 2017. Escherichia Coli ST131: a multidrug-resistant clone primed for global domination. F1000Res 6:F1000 Faculty Rev-195. doi:10.12688/f1000research.10609.1 PMC5333602 28344773

[29] Petty NK , Ben Zakour NL , Stanton-Cook M , Skippington E , Totsika M , Forde BM , Phan M-D , Gomes Moriel D , Peters KM , Davies M , Rogers BA , Dougan G , Rodriguez-Baño J , Pascual A , Pitout JDD , Upton M , Paterson DL , Walsh TR , Schembri MA , Beatson SA . 2014. Global dissemination of a multidrug resistant Escherichia coli clone. Proc Natl Acad Sci U S A 111:5694–5699. doi:10.1073/pnas.1322678111 24706808 PMC3992628

[30] Matsumura Y , Pitout JDD , Peirano G , DeVinney R , Noguchi T , Yamamoto M , Gomi R , Matsuda T , Nakano S , Nagao M , Tanaka M , Ichiyama S . 2017. Rapid identification of different Escherichia coli sequence type 131 Clades. Antimicrob Agents Chemother 61:e00179-17. doi:10.1128/AAC.00179-17 28584160 PMC5527616

[31] Johnson JR , Tchesnokova V , Johnston B , Clabots C , Roberts PL , Billig M , Riddell K , Rogers P , Qin X , Butler-Wu S , Price LB , Aziz M , Nicolas-Chanoine M-H , Debroy C , Robicsek A , Hansen G , Urban C , Platell J , Trott DJ , Zhanel G , Weissman SJ , Cookson BT , Fang FC , Limaye AP , Scholes D , Chattopadhyay S , Hooper DC , Sokurenko EV . 2013. Abrupt emergence of a single dominant multidrug-resistant strain of Escherichia coli. J Infect Dis 207:919–928. doi:10.1093/infdis/jis933 23288927 PMC3571447

[32] Roer L , Tchesnokova V , Allesøe R , Muradova M , Chattopadhyay S , Ahrenfeldt J , Thomsen MCF , Lund O , Hansen F , Hammerum AM , Sokurenko E , Hasman H . 2017. Development of a web tool for Escherichia coli Subtyping based on fimH Alleles. J Clin Microbiol 55:2538–2543. doi:10.1128/JCM.00737-17 28592545 PMC5527432

[33] Bailey LC , Forrest CB , Zhang P , Richards TM , Livshits A , DeRusso PA . 2014. Association of antibiotics in infancy with early childhood obesity. JAMA Pediatr 168:1063–1069. doi:10.1001/jamapediatrics.2014.1539 25265089

[34] Nobel YR , Cox LM , Kirigin FF , Bokulich NA , Yamanishi S , Teitler I , Chung J , Sohn J , Barber CM , Goldfarb DS , Raju K , Abubucker S , Zhou Y , Ruiz VE , Li H , Mitreva M , Alekseyenko AV , Weinstock GM , Sodergren E , Blaser MJ . 2015. Metabolic and metagenomic outcomes from early-life pulsed antibiotic treatment. Nat Commun 6:7486. doi:10.1038/ncomms8486 26123276 PMC4491183

[35] Hagberg L , Hull R , Hull S , Falkow S , Freter R , Edén CS . 1983. Contribution of adhesion to bacterial persistence in the mouse urinary tract. Infect Immun 40:265–272. doi:10.1128/iai.40.1.265-272.1983 6131870 PMC264844

[36] Roberts JA , Marklund BI , Ilver D , Haslam D , Kaack MB , Baskin G , Louis M , Möllby R , Winberg J , Normark S . 1994. The Gal(alpha 1-4)Gal-specific tip adhesin of Escherichia coli P-Fimbriae is needed for pyelonephritis to occur in the normal urinary tract. Proc Natl Acad Sci U S A 91:11889–11893. doi:10.1073/pnas.91.25.11889 7991552 PMC45341

[37] Bergsten G , Wullt B , Svanborg C . 2005. Escherichia coli, fimbriae, bacterial persistence and host response induction in the human urinary tract. Int J Med Microbiol 295:487–502. doi:10.1016/j.ijmm.2005.07.008 16238023

[38] Schreiber HL , Conover MS , Chou W-C , Hibbing ME , Manson AL , Dodson KW , Hannan TJ , Roberts PL , Stapleton AE , Hooton TM , Livny J , Earl AM , Hultgren SJ . 2017. Bacterial virulence phenotypes of Escherichia coli and host susceptibility determine risk for urinary tract infections. Sci Transl Med 9:eaaf1283. doi:10.1126/scitranslmed.aaf1283 28330863 PMC5653229

[39] Blyn LB , Braaten BA , White-Ziegler CA , Rolfson DH , Low DA . 1989. Phase-variation of Pyelonephritis-associated Pili in Escherichia coli: Evidence for transcriptional regulation. EMBO J 8:613–620. doi:10.1002/j.1460-2075.1989.tb03416.x 2656260 PMC400848

[40] Hernday A , Krabbe M , Braaten B , Low D . 2002. Self-perpetuating epigenetic pili switches in bacteria. Proc Natl Acad Sci U S A 99 Suppl 4:16470–16476. doi:10.1073/pnas.182427199 12202745 PMC139910

[41] Zamora M , Ziegler CA , Freddolino PL , Wolfe AJ . 2020. A thermosensitive, phase-variable epigenetic switch: pap revisited. Microbiol Mol Biol Rev 84:e00030-17. doi:10.1128/MMBR.00030-17 32727743 PMC7392537

[42] Stapleton AE , Stroud MR , Hakomori SI , Stamm WE . 1998. The globoseries glycosphingolipid sialosyl alactosyl globoside is found in urinary tract tissues and is a preferred binding receptor in vitro for uropathogenic Escherichia coli expressing pap-encoded adhesins. Infect Immun 66:3856–3861. doi:10.1128/IAI.66.8.3856-3861.1998 9673272 PMC108435

[43] Ambite I , Butler DSC , Stork C , Grönberg-Hernández J , Köves B , Zdziarski J , Pinkner J , Hultgren SJ , Dobrindt U , Wullt B , Svanborg C . 2019. Fimbriae reprogram host gene expression - divergent effects of P and type 1 fimbriae. PLoS Pathog 15:e1007671. doi:10.1371/journal.ppat.1007671 31181116 PMC6557620

[44] Schembri MA , Nhu NTK , Phan M-D . 2022. Gut-bladder axis in recurrent UTI. Nat Microbiol 7:601–602. doi:10.1038/s41564-022-01113-z 35505247

[45] Worby CJ , Schreiber HL , Straub TJ , van Dijk LR , Bronson RA , Olson BS , Pinkner JS , Obernuefemann CLP , Muñoz VL , Paharik AE , Azimzadeh PN , Walker BJ , Desjardins CA , Chou W-C , Bergeron K , Chapman SB , Klim A , Manson AL , Hannan TJ , Hooton TM , Kau AL , Lai HH , Dodson KW , Hultgren SJ , Earl AM . 2022. Longitudinal multi-Omics analyses link gut microbiome dysbiosis with recurrent urinary tract infections in women. Nat Microbiol 7:630–639. doi:10.1038/s41564-022-01107-x 35505248 PMC9136705

[46] Rogers BA , Sidjabat HE , Paterson DL . 2011. Escherichia coli O25B-St131: a pandemic, multiresistant, community-associated strain. J Antimicrob Chemother 66:1–14. doi:10.1093/jac/dkq415 21081548

[47] Paul S , Linardopoulou EV , Billig M , Tchesnokova V , Price LB , Johnson JR , Chattopadhyay S , Sokurenko EV . 2013. Role of homologous recombination in adaptive diversification of extraintestinal Escherichia coli. J Bacteriol 195:231–242. doi:10.1128/JB.01524-12 23123908 PMC3553836

[48] Qin J , Wilson KA , Sarkar S , Heras B , O’Mara ML , Totsika M . 2022. Conserved FimH mutations in the global Escherichia Coli ST131 multi-drug resistant lineage weaken Interdomain interactions and alter adhesin function. Comput Struct Biotechnol J 20:4532–4541. doi:10.1016/j.csbj.2022.08.040 36090810 PMC9428848

[49] Shaikh N , Martin JM , Hoberman A , Skae M , Milkovich L , McElheny C , Hickey RW , Gabriel LV , Kearney DH , Majd M , Shalaby-Rana E , Tseng G , Kolls J , Horne W , Huo Z , Shope TR . 2020. Biomarkers that differentiate false positive urinalyses from true urinary tract infection. Pediatr Nephrol 35:321–329. doi:10.1007/s00467-019-04403-7 31758242 PMC6942213

[50] Shaikh N , Shope TR , Hoberman A , Muniz GB , Bhatnagar S , Nowalk A , Hickey RW , Michaels MG , Kearney D , Rockette HE , Charron M , Lim R , Majd M , Shalaby-Rana E , Kurs-Lasky M , Cohen DM , Wald ER , Lockhart G , Pohl HG , Martin JM . 2020. Corticosteroids to prevent kidney scarring in children with a febrile urinary tract infection: a randomized trial. Pediatr Nephrol 35:2113–2120. doi:10.1007/s00467-020-04622-3 32556960 PMC7529851

[51] Johnson JR , Clermont O , Johnston B , Clabots C , Tchesnokova V , Sokurenko E , Junka AF , Maczynska B , Denamur E . 2014. Rapid and specific detection, molecular epidemiology, and experimental virulence of the O16 subgroup within Escherichia coli sequence type 131. J Clin Microbiol 52:1358–1365. doi:10.1128/JCM.03502-13 24501035 PMC3993632

[52] Krueger F . 2022. Trimgalore: a wrapper around cutadapt and Fastqc to consistently apply adapter and quality trimming to FastQ files, with extra functionality for RRBS data. Github. Available from: https://github.com/FelixKrueger/TrimGalore

[53] Bolger AM , Lohse M , Usadel B . 2014. Trimmomatic: A flexible trimmer for illumina sequence data. Bioinformatics 30:2114–2120. doi:10.1093/bioinformatics/btu170 24695404 PMC4103590

[54] Kim D , Song L , Breitwieser FP , Salzberg SL . 2016. Centrifuge: rapid and sensitive classification of metagenomic sequences. Genome Res 26:1721–1729. doi:10.1101/gr.210641.116 27852649 PMC5131823

[55] Bankevich A , Nurk S , Antipov D , Gurevich AA , Dvorkin M , Kulikov AS , Lesin VM , Nikolenko SI , Pham S , Prjibelski AD , Pyshkin AV , Sirotkin AV , Vyahhi N , Tesler G , Alekseyev MA , Pevzner PA . 2012. SPAdes: a new genome assembly algorithm and its applications to single-cell sequencing. J Comput Biol 19:455–477. doi:10.1089/cmb.2012.0021 22506599 PMC3342519

[56] Jolley KA , Bray JE , Maiden MCJ . 2018. Open-access bacterial population genomics: BIGSdb software, the pubmlst.org website and their applications. Wellcome Open Res 3:124. doi:10.12688/wellcomeopenres.14826.1 30345391 PMC6192448

[57] Ingle DJ , Valcanis M , Kuzevski A , Tauschek M , Inouye M , Stinear T , Levine MM , Robins-Browne RM , Holt KE . 2016. In silico serotyping of E. Coli from short read data identifies limited novel O-loci but extensive diversity of O:H serotype combinations within and between pathogenic lineages. Microb Genom 2:e000064. doi:10.1099/mgen.0.000064 28348859 PMC5343136

[58] Inouye M , Dashnow H , Raven L-A , Schultz MB , Pope BJ , Tomita T , Zobel J , Holt KE . 2014. SRST2: rapid genomic surveillance for public health and hospital microbiology labs. Genome Med 6:90. doi:10.1186/s13073-014-0090-6 25422674 PMC4237778

[59] Doumith M , Day M , Ciesielczuk H , Hope R , Underwood A , Reynolds R , Wain J , Livermore DM , Woodford N . 2015. Rapid identification of major Escherichia coli sequence types causing urinary tract and bloodstream infections. J Clin Microbiol 53:160–166. doi:10.1128/JCM.02562-14 25355761 PMC4290915

[60] Matsumura Y , Pitout JDD , Gomi R , Matsuda T , Noguchi T , Yamamoto M , Peirano G , DeVinney R , Bradford PA , Motyl MR , Tanaka M , Nagao M , Takakura S , Ichiyama S . 2016. Global Escherichia coli sequence type 131 clade with blaCTX-M-27 gene. Emerg Infect Dis 22:1900–1907. doi:10.3201/eid2211.160519 27767006 PMC5088012

[61] Jia B , Raphenya AR , Alcock B , Waglechner N , Guo P , Tsang KK , Lago BA , Dave BM , Pereira S , Sharma AN , Doshi S , Courtot M , Lo R , Williams LE , Frye JG , Elsayegh T , Sardar D , Westman EL , Pawlowski AC , Johnson TA , Brinkman FSL , Wright GD , McArthur AG . 2017. CARD 2017: expansion and model-centric curation of the comprehensive antibiotic resistance database. Nucleic Acids Res 45:D566–D573. doi:10.1093/nar/gkw1004 27789705 PMC5210516

[62] Li H , Durbin R . 2009. Fast and accurate short read alignment with burrows–Wheeler transform. Bioinformatics 25:1754–1760. doi:10.1093/bioinformatics/btp324 19451168 PMC2705234

[63] Hardiman CA , Weingarten RA , Conlan S , Khil P , Dekker JP , Mathers AJ , Sheppard AE , Segre JA , Frank KM . 2016. Horizontal transfer of carbapenemase-encoding plasmids and comparison with hospital epidemiology data. Antimicrob Agents Chemother 60:4910–4919. doi:10.1128/AAC.00014-16 27270289 PMC4958172

[64] Li H , Handsaker B , Wysoker A , Fennell T , Ruan J , Homer N , Marth G , Abecasis G , Durbin R . 1000. Genome project data processing subgroup. the sequence alignment/Map format and SAMtools.. Bioinformatics 25:2078–2079. doi:10.1093/bioinformatics/btp352 PMC2723002 19505943

[65] Paretree 1.0: Remove sequences, Bootstraps, and branch lengths from your trees. 2022. Retrieved 31 August 2022. Available from: http://emmahodcroft.com/PareTree.html

