# Supplementary material for: Genomic epidemiology and antibiotic susceptibility profiling of uropathogenic Escherichia coli among children in the United States
Source: mSphere. 2023 Aug 15;8(5):e00184-23. doi: 10.1128/msphere.00184-23 (PMC10597468; doi:10.1128/msphere.00184-23)
Supplement: Supplemental Figures and Table — Figures S1 to S7 and Table S8. [file msphere.00184-23-s0001.pdf]

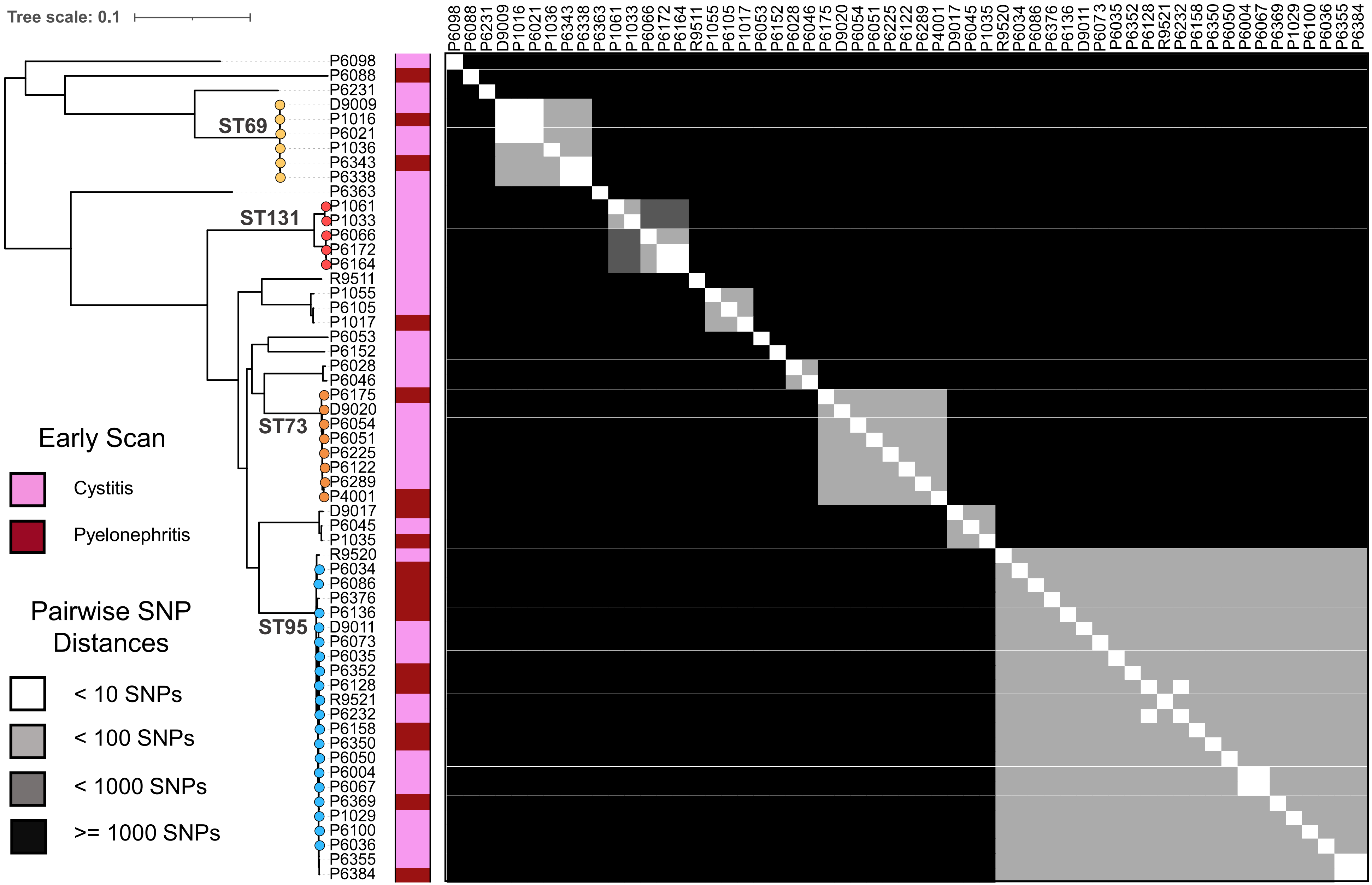

Figure S1: Diverse sequence-types, including four with at least five representatives (ST69, ST131, ST173, and ST95), were present in a sample of 57 children with urinary tract infection due to *E. coli*. A maximum-likelihood phylogeny showing the relationship between the 57 isolates is shown together with a greyscale heatmap displaying the number of SNP sites differentiating pairs of isolates (colored $\log_{10}$ scale). The colored circles on the leaves in the phylogeny correspond to the four most prevalent sequence types observed in the collection.

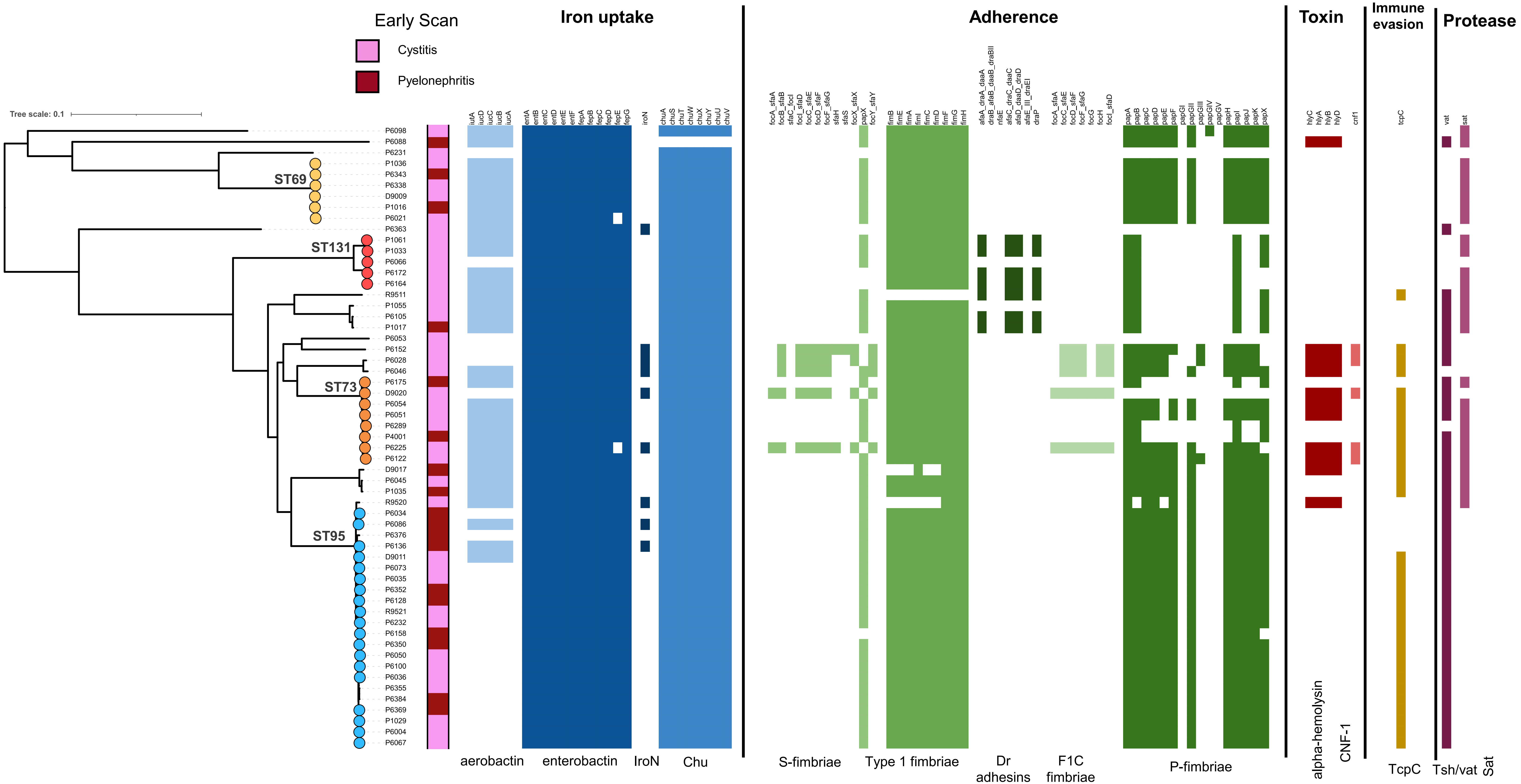

Figure S2: Presence of UPEC associated virulence genes in 57 isolates from children with UTIs. The carriage of specific virulence genes or alleles is shown across a phylogeny of the 57 isolates associated with cases of cystitis or pyelonephritis in our study. Virulence genes (columns) are grouped into blocks based on which virulence system or class they are associated with: iron uptake (blue), adherence (green), toxin (red), immune evasion (gold), protease (purple). Absence of alleles or genes is depicted as white.

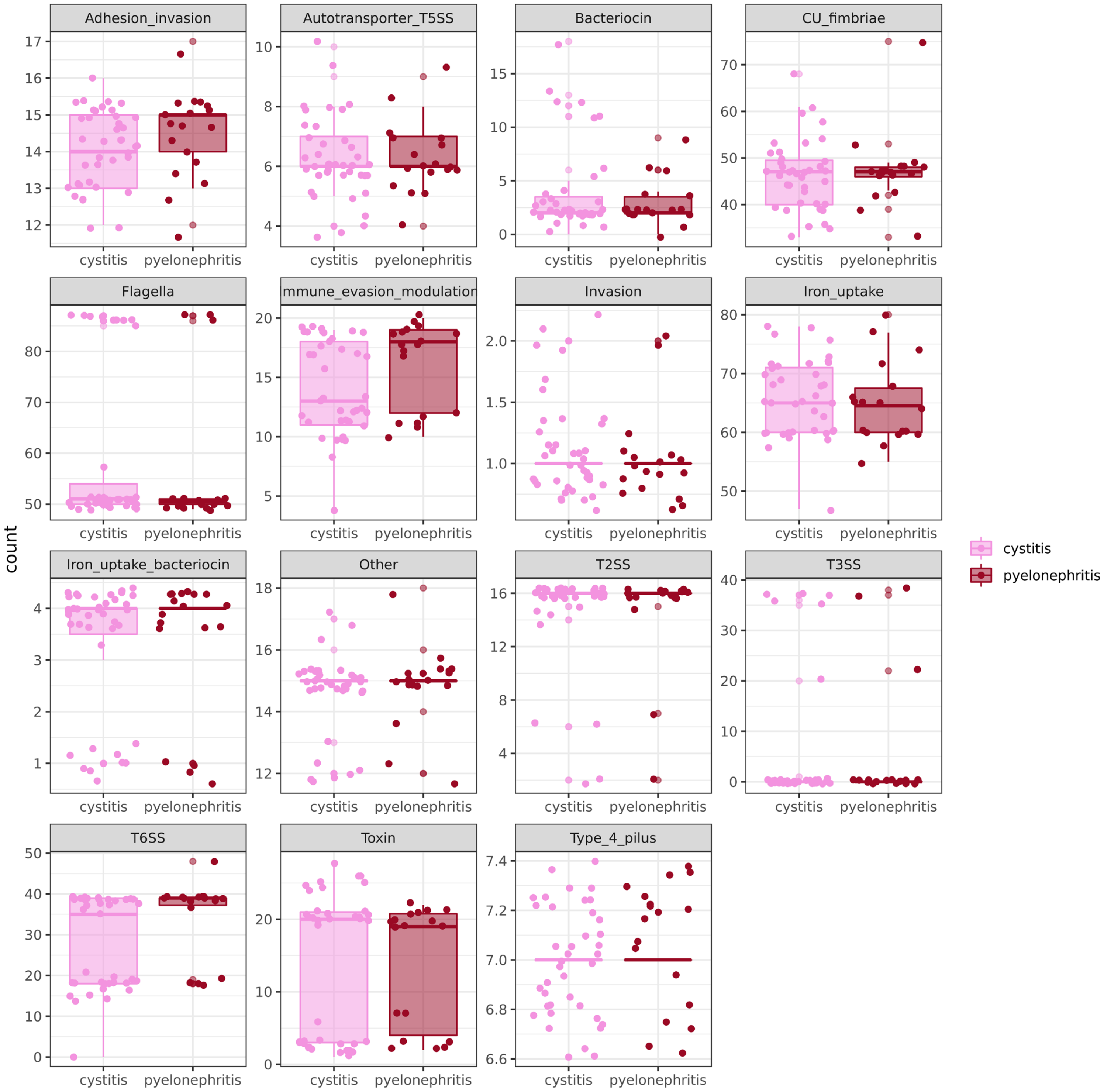

Figure S3: Counts of distinct genes in 14 virulence factor classes. Counts of distinct genes are shown for cystitis (n=39) and pyelonephritis (n=18) associated UPEC isolates.

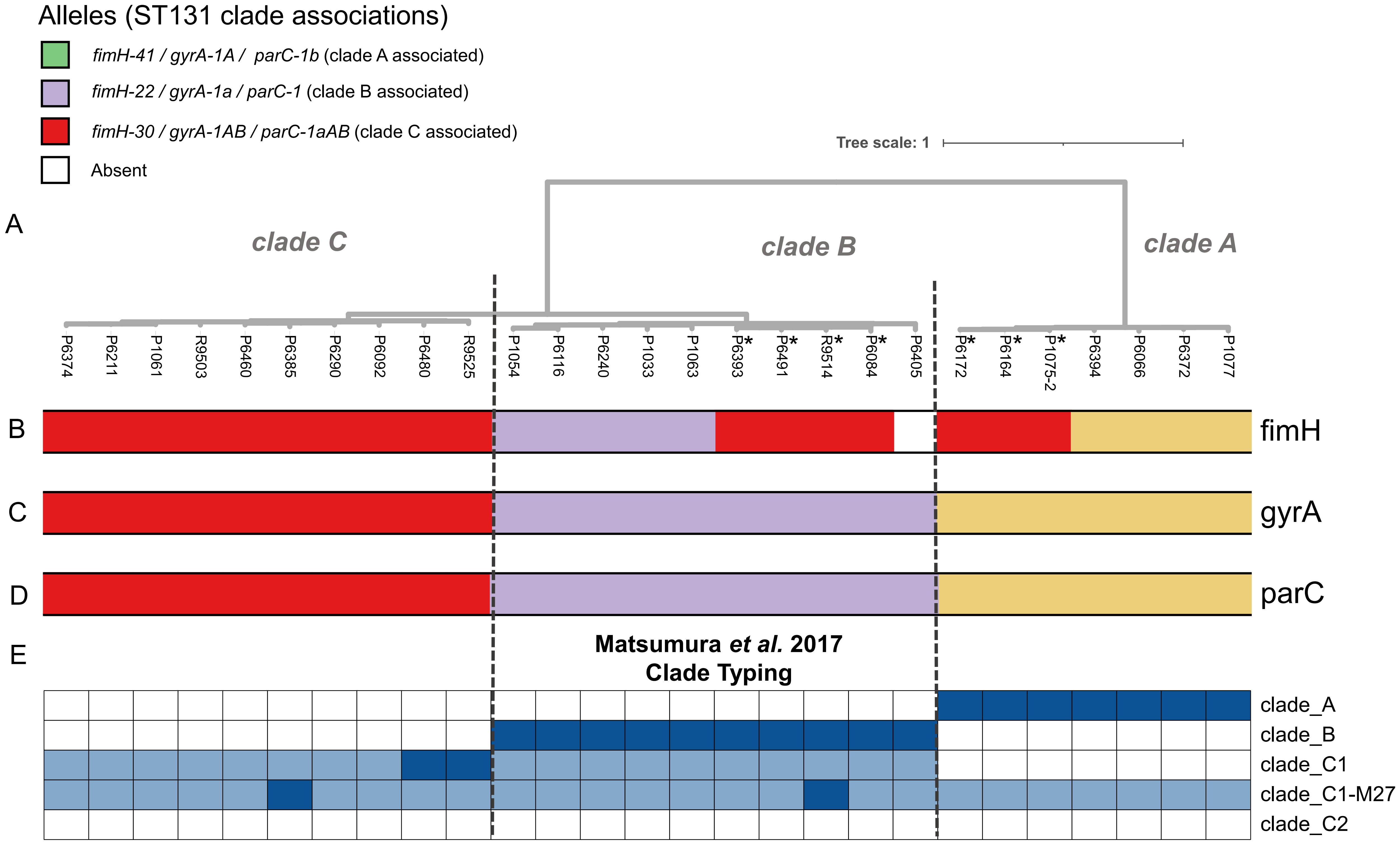

Figure S4: *fimH30* alleles show evidence of recombination. A) Maximum likelihood phylogeny of 27 ST-131 isolates. B) Distribution of *fimH* alleles. Isolates outside of clade C carrying *fimH30* are marked with asterisks (*). CD) To verify that the isolates were properly classified into clades, we confirmed that alternate genes used to distinguish clades, *parC* and *gyrA*, had typical clade-specific alleles. E) The presence of subclade specific primer sets in our sequencing reads is shown (Matsumura et al. 2017).

Supplemental Table S8: Presence of known genes contributing trimethoprim or sulfamethoxazole resistance (columns) across 27 ST131 isolates (rows).

CARD identifiers of antibiotic resistance genes are shown as columns with presence or absence of the gene for each isolate (rows) designated as 1 or 0, respectively.

Data is shown for a subset of genes, corresponding to different alleles of sul and drfA

[a] The MIC (μg/mL) for trimethoprim / sulfamethoxazole

Isolate / AMR Genes	SXT MIC[a]	Sulfamethoxazole		Trimethoprim Resistance Genes			
		sul1	sul2	dfrA1	dfrA8	dfrA12	dfrA17
P1061	>4/76	1	0	0	0	0	1
P1077	>4/76	1	0	0	0	0	1
P6164	>4/76	1	1	0	0	0	1
P6172	>4/76	1	1	0	0	0	1
P6211	>4/76	1	0	0	0	0	1
P6374	>4/76	1	0	0	0	0	1
P6385	>4/76	1	0	0	0	0	1
P6393	>4/76	1	0	0	0	1	0
P6405	>4/76	1	0	0	0	1	0
P6460	>4/76	1	0	0	0	0	1
P6480	>4/76	1	0	0	0	0	1
P6491	>4/76	1	0	0	0	1	0
R9514	>4/76	1	0	0	0	1	0
R9525	>4/76	1	1	0	0	0	1
P1033	≤2/38	0	0	0	0	0	0
P1054	≤2/38	0	0	0	0	0	0
P1063	≤2/38	0	0	0	0	0	0
P1075-2	≤2/38	0	0	0	0	0	0
P6066	≤2/38	0	0	0	0	0	0
P6084	≤2/38	0	0	0	0	0	0
P6092	≤2/38	0	0	0	0	0	0
P6116	≤2/38	0	0	0	0	0	0
P6240	≤2/38	0	0	0	0	0	0
P6290	≤2/38	0	0	0	0	0	0
P6372	≤2/38	0	0	0	0	0	0
P6394	≤2/38	0	0	0	0	0	1
R9503	≤2/38	0	0	0	0	0	0
